# Mycophilic or Mycophobic? Legislation and Guidelines on Wild Mushroom Commerce Reveal Different Consumption Behaviour in European Countries

**DOI:** 10.1371/journal.pone.0063926

**Published:** 2013-05-21

**Authors:** Ursula Peintner, Stefanie Schwarz, Armin Mešić, Pierre-Arthur Moreau, Gabriel Moreno, Philippe Saviuc

**Affiliations:** 1 Institute of Microbiology, University Innsbruck, Innsbruck, Austria; 2 Institut Ruđer Bošković, Zagreb, Croatia; 3 Commission Environnement de la Société Mycologique de France, Paris, France; 4 Laboratoire des Sciences Végétales et Fongiques, UFR Pharmacie, Université Lille Nord de France, Lille, France; 5 Departamento Ciencias de la Vida (Botánica), Universidad de Alcalá, Alcalá de Henares, Madrid, Spain; 6 Toxicovigilance Center, Michallon Hospital, Grenoble, France; Institut de Genetique et Microbiologie, France

## Abstract

Mycophiles forage for and pick vast quantities of a wide variety of wild mushroom species. As a result, mushroom intoxications are comparatively frequent in such countries with mycophiles. Thus, national governments are forced to release guidelines or enact legislation in order to ensure the safe commerce of wild mushrooms due to food safety concerns. It is in these guidelines and laws that one can observe whether a country is indeed mycophobic or mycophilic. Furthermore, these laws and guidelines provide valuable information on mushroom preferences and on the consumption habits of each country. As such we were interested in the questions as to whether mushroom consumption behaviour was different within Europe, and if it was possible to discover the typical or distinctive culinary preferences of Slavic or Romanic speaking people, people from special geographical regions or from different zones. This work is based on the analysis of edible mushroom lists available in specific guidelines or legislation related to the consumption and commerce of mushrooms in 27 European countries. The overall diversity of edible mushrooms authorised to be commercialised in Europe is very high. However, only 60 out of a total 268 fungal species can be cultivated. This highlights the importance of guidelines or legislation for the safe commerce of wild mushrooms. The species richness and composition of the mushrooms listed for commerce is very heterogeneous within Europe. The consumption behaviour is not only language-family-related, but is strongly influenced by geographical location and neighbouring countries. Indicator species were detected for different European regions; most of them are widespread fungi, and thus prove culture-specific preferences for these mushrooms. Our results highlight tradition and external input such as trade and cultural exchange as strong factors shaping mushroom consumption behaviour.

## Introduction

Mushrooms are a prised food in certain regions of the world, but are approached with suspicion in others. For example, there is a long history of collecting and eating wild mushrooms in countries and regions such as Southeast Asia, the Venezuelan Amazon, in Slavic countries and in Italy. The population of these countries are especially fond of mushrooms, and have therefore been labelled as mycophile [1], [2], [3]. On the other hand, mushrooms are rarely picked and consumed in the United Kingdom [2]. Therefore, it was not surprising that a British mycologist, namely W.D. Hay introduced the term mycophobia (later fungophobia) in 1887. Mycophobia is the fear of mushrooms and fungi. Fiction, including Lewis Carroll's Alice in the Wonderland (1865), H.G. Walls's The Purple Pileus (1895) and Brian Lumley's Fruiting Bodies (1988) have further promoted mycophobia. But are these rumours about “the mycophobic Germans” or “the mycophilic Italians” prejudices, or are they based on a real mycophobe or mycophile attitude of the population? A mycophile person is one whose hobby is hunting and foraging for wild edible mushrooms. Hunting for wild mushrooms also implies their consumption and circulation. Unfortunately, mushroom intoxications are a frequent consequence of profuse mushroom picking and consumption by mycophiles. In Northern Italy for instance, the commerce of fresh and preserved wild mushrooms was extremely important in the 18^th^ century, and caused many cases of mycetism every year. In response to this problem, the first important set of rules concerning the commerce of wild fungi was elaborated in 1820 under the Austrian-Hungarian domination, with successive additions and modifications in 1823 and 1856 [4]. Other countries followed suit and either released guidelines or enacted laws concerning the consumption and commercialisation of mushrooms. Apart from providing lists of wild fungal taxa, which can be commercialised in the country, mushroom legislation also often controls the procedure for collecting wild mushrooms (e.g. time, quantities, allowed methods), and thus also incorporates environmental conservation measures.

Our basic assumption was that countries with a mycophilic population had specific guidelines or legislation concerning the marketing of wild mushrooms, which include a comparatively large number of mushroom species; whereas countries with a mycophobic population, either had guidelines or legislation including very few mushroom species, or covered the risk posed from all food groups, which includes mushrooms brought to the market, by the EU General Food Law – general legislation which bans food harmful to the consumer.

Harvesting and marketing wild food, including mushrooms, is of a growing interest in most countries now. We were therefore especially interested in the question if and how mushroom consumption behaviour differs between European countries, and if these differences are culture-related. We approached these questions by analysing and comparing guidelines or legislation concerning the commercialisation of mushrooms in European countries. Our investigation has enormous implications on mushroom guidelines and legislation; this is because mushroom consumption behaviour has been addressed in a large geographical context for the first time. We detected significant differences in mushroom culinary traditions within Europe. They are clearly related to culture in a geographical context, and are strongly influenced by the region due to trade and cultural exchanges.

## Materials and Methods

This work is based on the analysis of guidelines or legislation dealing with the commercialisation of mushrooms in 42 European countries. They were either retrieved directly from the World Wide Web, or requested from resident mycologists based in the respective European country: These individuals provided the requested information, or informed us that guidelines or legislation were not available in their country (Table S1); unfortunately, it was not possible to obtain information from all European countries (Table 1).

**Table 1:** European countries listing mushroom species for commercialisation: countries with legislation, guidance lists, without lists, or with no information.

Legislation (16)	Guidelines (7)	No lists** (11)	No information (12)
Austria	Belgium	Bulgaria	Albania
Belarus	Portugal	Estonia	Andorra
Bosnia and Herzegovina	Ukraine	Germany	Czech Republic
Croatia		Greece	Cyprus
Finland*		Hungary	Kosovo
France		Ireland	Liechtenstein
Italy		Lithuania	Luxemburg
Macedonia		Latvia	Malta
Montenegro	Nordic co-operation	Netherlands	Moldavia
Poland	Denmark	Slovenia	Monaco
Rumania	Finland	United Kingdom	San Marino
Russia	Iceland		Turkey
Serbia	Norway		
Slovakia	Sweden		
Spain	Faroe Islands		
Sweden	Greenland		
Switzerland	Åland		

* List valid until 1.7.2012, now EVIRA (Finnish Food Safety Authority Evira) only makes recommendations of the species (the list is about the same as the earlier list), which can go to market, but all edible mushrooms can be on sale. Moreover, also the guidelines of the Nordic Co-operation cover Finland.

** Some countries have legislation concerning mushroom picking and nature conservation.

European mushroom-specific regulations are either guideline or legislation based on traditional, mycological background, and on risk assessment. Mushroom legislation is national e.g. in Austria, Belarus, Poland, Russia, Slovakia, Spain, Switzerland etc. Italy has a national list, used in conjunction, with additional regional lists for departments or political regions, accounting for the local culinary preferences of the population. France's current legislation is decentralised, the list presented here is based on prefectoral orders (8 departments) and municipal orders (43 municipalities) [5] applying to fungi, which are authorised to be sold in markets. A proposed national list was prepared in 2010 by the French Mycological Society on request of the General Directorate for Competition, Consumer Affairs and Prevention of Fraud (DGCCRF), this proposed list is still under consultation. Spanish mushroom legislation is special as it not only contains lists of wild and cultivated mushrooms permitted for commerce in fresh conditions (some only after having received a special treatment), but also a list of mushroom species whose commerce is strictly forbidden. Additional notes and information have been published before [6]. Recently, the “Nordic Co-operation” (Denmark, Finland, Iceland, Norway, Sweden, Faroe Islands, Greenland, Åland Islands) released a common guideline on mushrooms traded as food [7], [8]. In the Ukraine, the ministry of health is currently working on detailed rules and legislation. However, for the moment there is no official document about the mushroom trade at a national level. The Ukrainian list of species is based on a publication about edible mushrooms used for consumption in Ukraine [9]. In addition, it includes recent amendments provided by Ukrainian mycologists. In Portugal, edible mushroom species lists are being compiled too: the lists of edible mushrooms were provided based on a conservative estimation of the current use in the country [10].

In countries of former Yugoslavia, Croatia was the first country to regulate the exploitation of wild mushrooms for commercial use. Other countries mostly followed Croatian example, but they adapted the regulations according to their specific local situation. With the exception of Slovenia, all the countries (Bosnia and Herzegovina, Croatia, Macedonia, Montenegro, Serbia) have included the lists of the species of mushrooms that are allowed for commercialisation in their legislature.

Finally, a list of all edible mushroom taxa authorised to be sold commercially in Europe was compiled based on 22 European lists. Mushroom names were carefully revised concerning synonymies and different genus attributions (e.g. *Xerocomus badius* or *Boletus badius*) (Table S2, Table S3). Mushroom species complexes (e.g. *Armillaria mellea* s.l.) were used in a conservative way, and counted as one cumulative species. The same conservative procedure was applied for cases where different, not explicitly named species of a genus (e.g. *Helvella* spp.) were listed.

There are significant differences in the composition of European country mushroom lists, as some countries, e.g. Switzerland, list mushroom species, which are nowadays mostly cultivated together with wild mushroom species, whilst other countries, such as Croatia and France, list only wild edible species. However, only 22% of all edible mushroom species are cultivated fungi; moreover these were found not to significantly influence our analyses, and where therefore included in the list for the sake of thoroughness.

PC-ORD 6 [11] was used to compare mushroom lists. Outlier analyses and Multi-Response Permutation Procedures (MRPPs) were carried out using Euclidean distances. Indicator species analysis was carried out with the method of Dufrene and Legendre [12]. Nonmetric Multidimensional Scaling (NMS) was done with Sorensen (Bray-Curtis) distances, 6 axes, a maximum of 500 iterations in the autopilot mode. Country groups were defined based on three criteria: a) Language family (Romance = France, Italy, Portugal, Romania, Spain; Slavic = Belarus, Bosnia and Herzegovina, Croatia, Macedonia, Montenegro, Poland, Serbia, Slovakia, Ukraine, Russia; Finno-Ugric = Finland; Germanic = Austria, Nordic Co-operation, Sweden; Mixed = Belgium, Switzerland); b) Geography (Southwest Europe = France, Italy, Portugal, Spain; Southeast Europe = Bosnia and Herzegovina, Croatia, Macedonia, Montenegro, Serbia, Republika Srpska; Central Europe = Austria, Belgium, Switzerland; Eastern Europe = Belarus, Poland, Romania, Russia, Slovakia, Ukraine; Nordic Co-operation = Finland, Nordic Co-operation, Sweden); c) Neighbouring countries (West = Austria, Belgium, France, Italy, Portugal, Spain, Switzerland; Nordic = Nordic Co-operation, Sweden; East = Belarus, Finland, Poland, Russia, Romania, Slovakia, Ukraine; Former Yugoslavia = Bosnia and Herzegovina, Croatia, Macedonia, Montenegro, Serbia, Republika Srpska). A goodness-of-fit test for normal distribution was carried out using the Kolmogorov-Smirnov- test for each dataset. To analyse the effect of language family, geography, and neighbouring countries, significant differences (p<0.05) were tested with ANOVA, Tukey HSD test for normal distributed datasets. All statistical tests were carried out with STATISTICA 9.1., StatSoft, Inc. (2010) www.statsoft.com.

## Results and Discussion

### Mushroom legislation or guidelines reflect a mycophilic or mycophobic attitude

More than half (23) of 46 European countries have guidelines or legislation concerning the consumption and commercialisation of mushrooms. Eleven countries do not have mushroom-specific guidelines or legislation, although some of them have legislation concerning mushroom picking and nature conservation. No information was available from twelve countries (Table 1). When only considering countries, which we have information from, 67% of them specifically regulate wild mushroom commerce.

**Figure 1:**
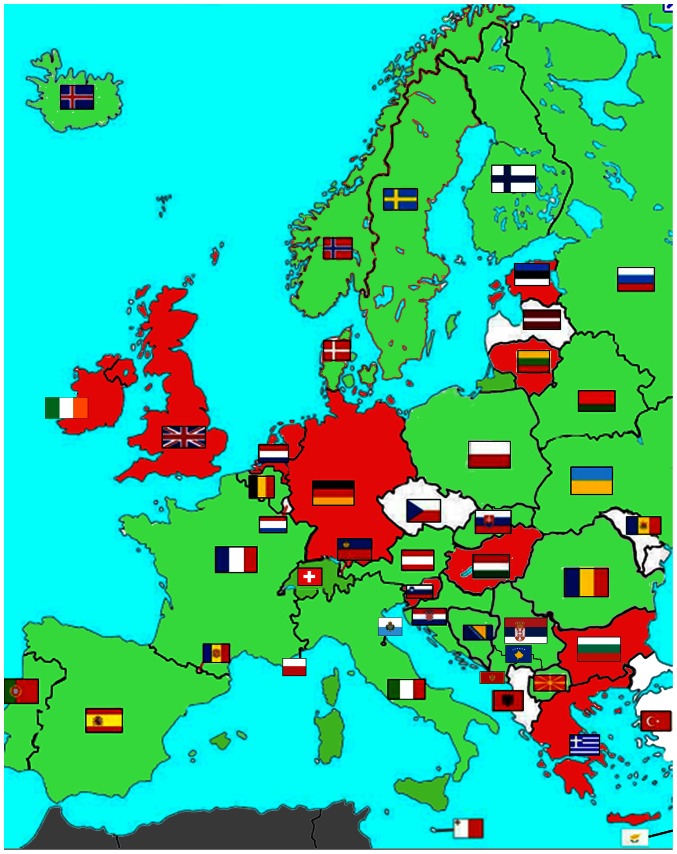
Map of Europe. European countries with mushroom legislation or guidelines (green), without them (red), or with no information available (white).

At first glance, it is striking that some countries with a Germanic-speaking population (Dutch, English, German) (Figure 1) do not usually have specific mushroom guidelines or legislation. This indicates a mycophobic attitude of the population in these countries. Austria is one exception: We speculate that the influence of e.g. the Italian neighbours collecting, selling and marketing mushrooms in Austria could have triggered the enactment of mushroom legislation. Furthermore, strong historical ties between Austria and Northern Italy could be another reason. Scandinavia (Denmark, Norway and Sweden) has also a Germanic speaking population: these countries have had mushroom guidelines or advice for some mushroom species for several years, and have only recently released a common mushroom guideline with other Nordic countries [7], [8]. Germanic-speaking countries have generally been considered to have a mycophobic population [2]; mushroom picking and commercialisation are not an issue of public interest in such countries, as indigenous people only consume a few mushroom species and, most of them are cultivated.

Western European countries with mushroom legislation or guidelines are countries with a Romanic-speaking population (France, Italy, Portugal, Spain) or with Romanic-speaking minorities (Belgium, Switzerland). Most Eastern European countries with mushroom legislation or guidelines have a Slavic-speaking population (Belarus, Poland, Russia, Slovakia, Ukraine, countries from former-Yugoslavia). This suggests that the mycophilic attitude of the population could be culture-related, and thus be typical for Romanic and Slavic-speaking people. Indeed, mushrooms and their consumption are of a huge economic importance in countries with a mycophilic population. Mycophilic indigenous people collect and consume large quantities of many different species of wild mushrooms. Mushrooms are collected for recreation, they are freshly prepared or preserved in different ways, and they are sold or given away as a treasured gift [1]. In consequence, such countries are obliged to release public guidelines for the commercialisation of wild mushrooms due to food safety issues.

### The diversity of edible mushrooms commercialised in Europe is very high

**Figure 2:**
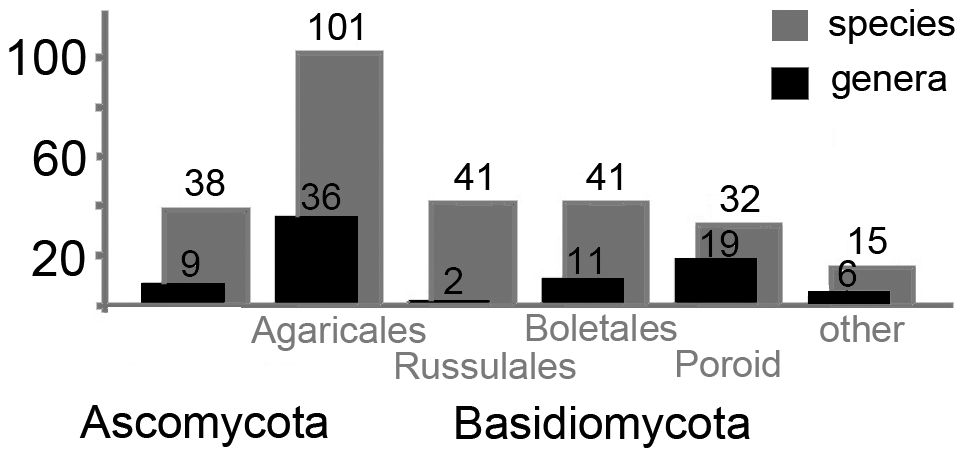
Distribution of edible mushrooms to different groups of Ascomycota and Basidiomycota. The Basidiomycota are subdivided into Agaricales, Russulales, Boletales, Poroid Fungi and other groups of Basidiomycota (n = 268 species in 83 genera).

The diversity of mushroom species commercialised in Europe is amazingly high: a total of 268 fungal taxa are listed fit to be commercialised in 24 European countries (282 when also considering the new list proposed for France) (Table S2, Table S3). The lists include nine genera of Ascomycota and 74 genera of Basidiomycota. The Agaricales are the group represented by the largest number of genera (36) and species (>102). In contrast, the Russulales consist of two genera only, but are represented by a large number of species (42) (Figure 2).

Only 60 of all the listed fungal taxa can be cultivated in commercial mushroom farms; all other species are wild mushrooms collected by mushroom pickers and sold on the market. This highlights the importance of guidelines or legislation for the safe commercialisation of wild mushrooms.

### Mushroom guidelines or legislation are different in European countries

**Figure 3:**
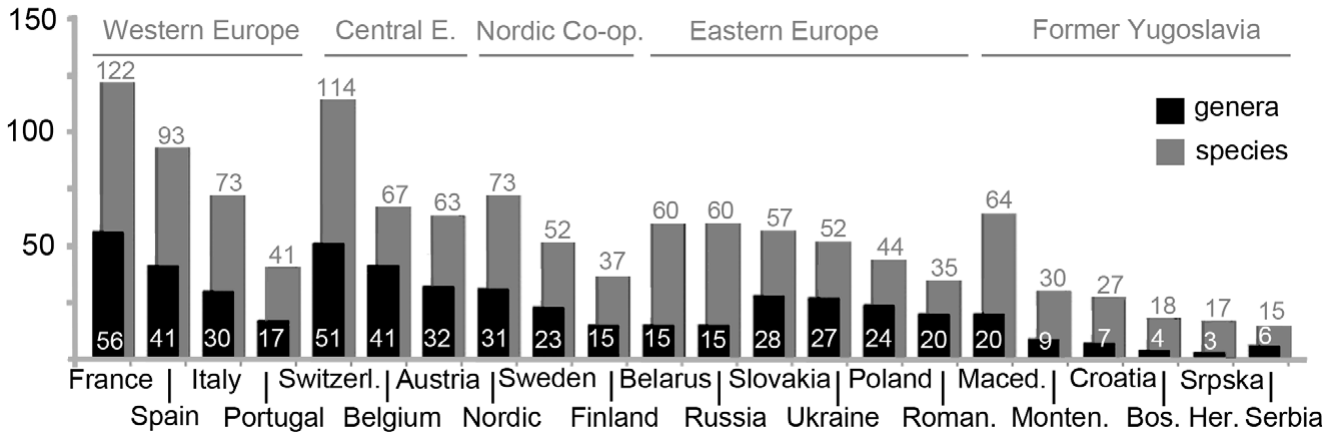
Species- and genus richness of edible mushrooms in 21 European countries and the Nordic Co-operation. Data are based on lists of specific mushroom legislation or guidelines.

Mushroom lists published by European countries differ widely with respect to their comprehensiveness: they range from a minimum of 15 listed species (Serbia) to a maximum of 122 (compiled list France) (Figure 3). Switzerland and Spain have the most comprehensive national lists. Over comprehensive lists are often confusing and difficult to administer: this was one reason why Switzerland reduced the number of species on the list from to 142 to 114 in 2012. Italy has a national list of 73 species, regional additions bring the overall number to around 150 species [4]. Besides these frontrunner countries in mushroom diversity, most European countries allow for the commercialisation of about 55 mushroom species (MW = 55, SD = 29, Median = 55).

European mushroom lists are also very heterogeneous when considering genera: they allow for the commercialisation from three (Republika Srpska) to 56 (France) fungal genera (MW = 23, SD = 14, Median = 21). *Lactarius* (23 spp.), *Russula* (19 spp.) and *Agaricus* (14 spp.) are the genera with most species authorised for commercialisation (Table S2).

However, species composition is especially very heterogeneous within European mushroom guidelines and legislation: all together, 268 mushroom taxa are listed in 83 genera (Table S2), but only two fungal taxa are on all the lists: Porcini mushrooms (*Boletus edulis* complex), and Chanterelle (*Cantharellus cibarius*). These absolute market leaders are widespread and important non-timber-forest-products, and are commercially harvested throughout the whole world [4], [13], [14], [15], [16]. Furthermore, the Saffron Milkcap (*Lactarius deliciosus*), Morels (*Morchella esculenta*), the Bay Bolete (*Boletus badius*), Field Mushrooms (*Agaricus campestris*), and the Black Trumpet (*Craterellus cornucopioides*) are authorised for marketing in >70% of European countries (Table S2). The global trade of these “top sellers” offers a significant income to rural producers and processors around the globe.

In contrast, only half (134) of all the edible mushroom species are authorised to be commercialised in only in one or two European countries. This indicates that local tradition, predilection, and mushroom taste are very different throughout Europe. But are these culinary differences culture-related (e.g. related to language groups), due to geographical reasons (e.g. different climate, distribution of plants and their related fungi), due to influence from neighbouring countries (trade), or just random?

**Figure 4:**
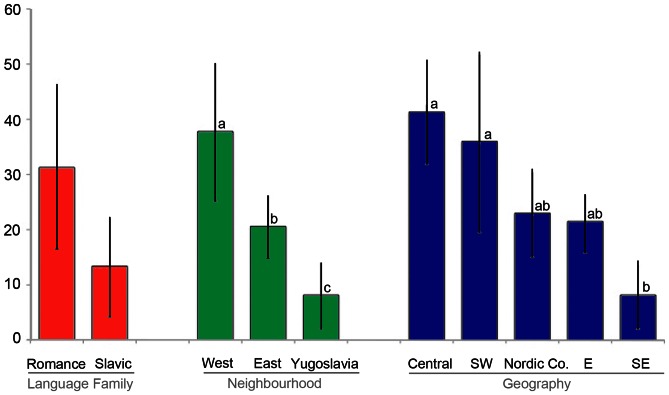
Richness of edible mushroom genera (MW, SD) in different groups of European countries. Groups were made based on Language family, Geography and Neighbouring countries. Language family (Romance = France, Italy, Portugal, Romania, Spain; Slavic = Belarus, Bosnia and Herzegovina, Croatia, Macedonia, Montenegro, Poland, Serbia, Slovakia, Ukraine, Russia); Geography (Central Europe = Austria, Belgium, Switzerland; Southwest Europe = France, Italy, Portugal, Spain; Nordic Co-operation = Finland, Nordic Co-operation, Sweden; Eastern Europe = Belarus, Poland, Romania, Russia, Slovakia, Ukraine; Southeast Europe = Bosnia and Herzegovina, Croatia, Macedonia, Montenegro, Serbia, Republika Srpska); Neighbouring countries (West = Austria, Belgium, France, Italy, Portugal, Spain, Switzerland, Nordic = Nordic Co-operation, Sweden; East = Belarus, Finland, Poland, Russia, Romania, Slovakia, Ukraine. Former Yugoslavia = Bosnia and Herzegovina, Croatia, Macedonia, Montenegro, Serbia, Republika Srpska. a, b: significant differences.

**Figure 5:**
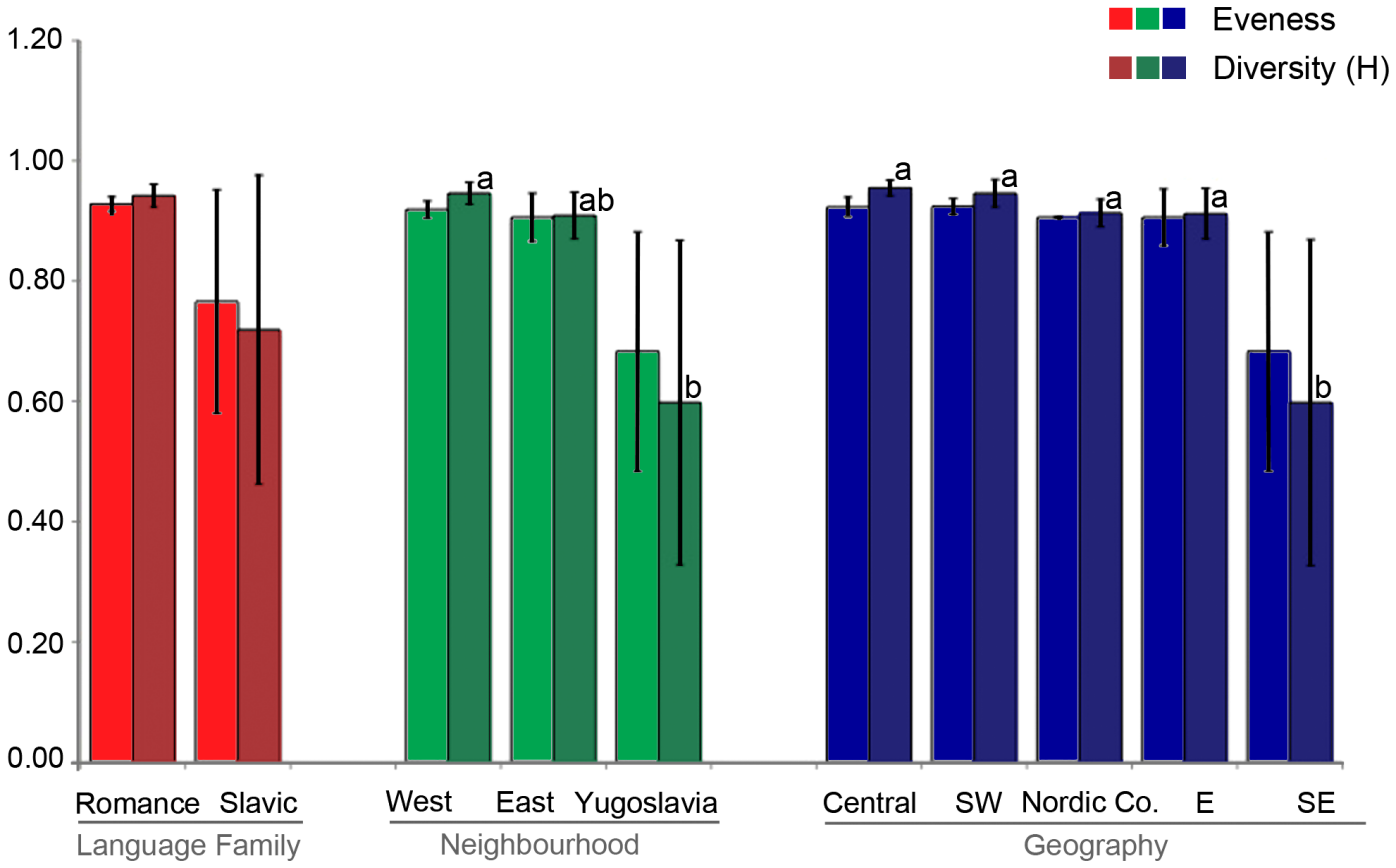
Eveness and diversity (Simpson's D) of edible mushroom genera (MW, SD) in Europe. Groups were made based on Language family, Geography and Neighbouring countries. Language family (Romance = France, Italy, Portugal, Romania, Spain; Slavic = Belarus, Bosnia and Herzegovina, Croatia, Macedonia, Montenegro, Poland, Serbia, Slovakia, Ukraine, Russia); Geography (Central Europe = Austria, Belgium, Switzerland; Southwest Europe = France, Italy, Portugal, Spain; Nordic Co-operation = Finland, Nordic Co-operation, Sweden; Eastern Europe = Belarus, Poland, Romania, Russia, Slovakia, Ukraine; Southeast Europe = Bosnia and Herzegovina, Croatia, Macedonia, Montenegro, Serbia, Republika Srpska); Neighbouring countries (West = Austria, Belgium, France, Italy, Portugal, Spain, Switzerland, Nordic = Nordic Co-operation, Sweden; East = Belarus, Finland, Poland, Russia, Romania, Slovakia, Ukraine. Former Yugoslavia = Bosnia and Herzegovina, Croatia, Macedonia, Montenegro, Serbia, Republika Srpska. a, b: significant differences.

The most mycophilic Europeans live in the Southwest- and in Central Europe, and that they are predominantly Romance-speaking. This initial hypothesis was confirmed by the quantitative analyses of European mushroom lists: Mushroom markets in the west of Europe are significantly more species rich and diverse than those in the east of Europe; and the latter are more diverse than mushroom markets in former-Yugoslavian countries. These quantitative factors were also statistically significant when focussing on geographically distinct regions: Countries in SE Europe have significantly less mushroom species and a lower diversity authorised for commerce than countries in Central- and SW Europe, but differences to Eastern Europe and the Nordic Co-operation were not significant. Lists from Slavic speaking countries were less diverse than from Romance speaking countries, but these differences were not significant (Figures 4–5).

When considering qualitative aspects, we found that geographical location and the influence of neighbouring states were very strong drivers for the species composition of lists for mushroom commerce (Table 2): People from neighbouring states in the former Yugoslavia, as well as from neighbouring states in the East and in the West of Europe have, significantly different mushroom preferences to one another. Trade and cultural exchange influenced the consumption behaviour: this is also shown by the fact that Central European countries (Belgium, Switzerland and Austria) do no not differ significantly from their neighbouring states in SW and Eastern Europe, but they clearly differ from non neighbouring countries (Nordic Co-operation, SE Europe). Local taste and tradition, as well as commerce with direct neighbours are stronger drivers than climate and vegetation type: country groups with similar climate (SW vs. SE Europe or Eastern Europe vs. Nordic Co-operation) and a similar occurrence of mushroom species have significantly different mushroom traditions.

**Table 2:** Significant differences in species composition (based on MRPP) of European lists of edible mushrooms for different language families, for neighbouring countries, and for geography.

Category	Pairwise Comparison of Groups	p	*
Geography	Central Europe vs. Nordic Co-operation	0.0227	*
	Central vs. Eastern Europe	0.0510	
	Central vs. SE Europe	0.0046	**
	Central vs. SW Europe	0.1610	
	Eastern Europe vs. Nordic Co-operation	0.0497	*
	Eastern vs. SE Europe	0.0005	***
	Eastern vs. SW Europe	0.0108	*
	SW Europe vs. Nordic Co-operation	0.0122	*
	SW vs. SE Europe	0.0025	**
Language familiy	Germanic vs. Mixed	0.0000	***
	Germanic vs. Romance	0.0597	
	Germanic vs. Slavic	0.0774	
	Romance vs. Mixed	0.5421	
	Slavic vs. Mixed	0.0854	
	Slavic vs. Romance	0.0238	*
Neighbouring	Eastern vs. Former Yugoslavia	0.0005	***
	Western vs. Eastern	0.0008	***
	Western vs. Former Yugoslavia	0.0001	***

* p≤0.05;

** p≤0.005;

*** p≤0.0005.

MRPP for geography = Central-, Eastern-, SW-Europe, SE-Europe, Scandinavia; A = 0.1541, p = 0.0000 ***.

MRPP for language families = Germanic, Mixed, Romance, Slavic: A = 0.0595, p = 0.0180 *.

MRPP for neighbouring countries = West, East, Former Yugoslavia: A = 0.1145, p = 0.0000 ***.

**Figure 6:**
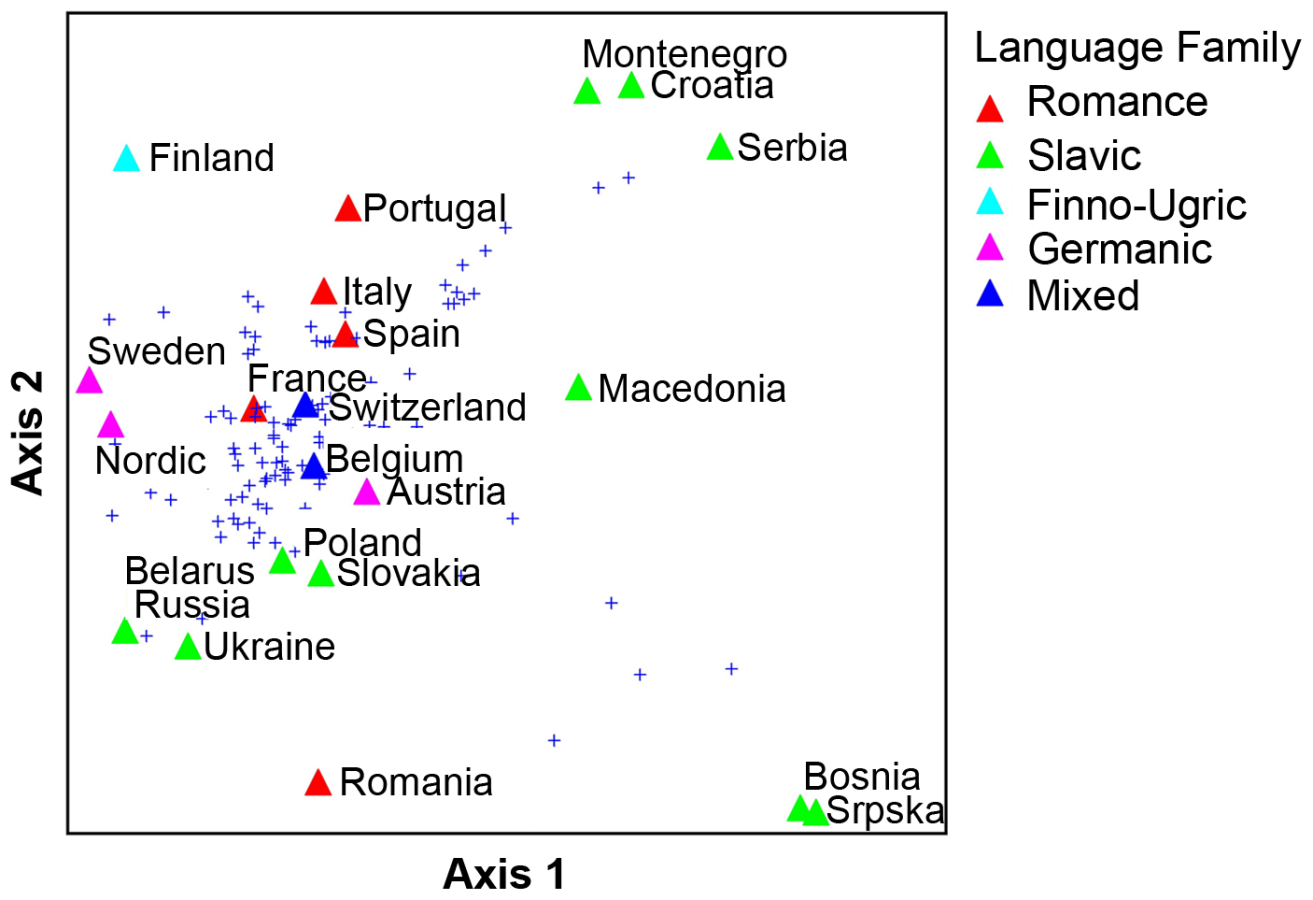
NMS ordination of mushroom species commercialised in 21 European countries and the Nordic Co-operation. Language groups are coded with different colours. (NMS Distance measure: Sorensen, random starting, 500 iterations, 250 with real data, 9.85462 = final stress for a 2-dimensional solution. Axis 1: 63.3% of variance based on r^2^, Axis 2: 19.3% of variance).

Language group was also a significant factor shaping mushroom preferences, but was dependent on geography and proximity: (Tables 2–3). Countries with Romance-speaking populations generally have different mushroom preferences than the Slavic speaking European populations, but differences blur with mixed-speaking countries, and mushroom taste in Romania is strongly influenced by the Slavic-speaking northern neighbours (Figure 6). Countries with Slavic-speaking population form two groups with different mushroom tastes and traditions: the Eastern European countries and countries from the former Yugoslavia.

**Table 3:** Significant differences in genus composition (based on MRPP) of European mushroom lists for geography and for neighbouring countries.

Category	Pairwise Comparison of Groups	p	*
Geography	Central vs. Eastern Europe	0.2143	
	Central vs. Nordic Co-operation	0.0361	*
	Central vs. SE Europe	0.0476	*
	Central vs. SW Europe	0.1771	
	Eastern Europe vs. Nordic Co-operation	0.1990	
	Eastern vs. SE Europe	0.0013	**
	Eastern vs. SW Europe	0.0303	*
	Nordic Co-operation vs. SW Europe	0.0323	*
	SE Europe vs. Nordic Co-operation	0.0084	*
	SE vs. SW Europe	0.0098	*
Neighbouring countries	West vs. East	0.0125	
	West vs. Former Yugoslavia	0.0000	***
	East vs. Former Yugoslavia	0.0007	***

* p≤0.05;

** p≤0.005;

*** p≤0.0005.

MRPP for Geography = Central-, Eastern-, SW-Europe, SE-Europe, Nordic Co-operation: A = 0.15688418, p = 0.0002 ***.

MRPP for Neighbouring countries = West, East, Former Yugoslavia: A = 0.1259, p = 0.0001 ***.

We concluded that Europe harbours at least four different traditions related to the consumption of edible mushrooms. One mycophobic tradition (Germanic-speaking countries), as well as several mycophilic traditions with different mushroom predilections: one group of Romance-speaking countries in Western Europe, two groups of predominantly Slavic-speaking people in Eastern Europe, and the Nordic Co-operation countries. The significant differences in mushroom taste between the populations of these countries are due to geographical separation, e.g. by mycophobic countries, and due to language and cultural barriers. This has enormous implications, as it highlights the cultural significance and the influence of external input on mushroom consumption behaviour: people speaking different language groups value different edible mushroom species for many different reasons. Culture has also been shown to be a crucial factor shaping traditional mycological knowledge in Mexico: the Zapotec indigenous population attribute different fungal species with different values in taste, food use, health and economy [14].

### The different consumption behaviour in European countries of wild mushrooms

The population of different European regions differs clearly in their consumption behaviour of wild mushrooms (Figure 6) (NMS Distance measure: Sorensen, random starting, 500 iterations, 250 with real data, 9.85462 = final stress for a 2-dimensional solution. Axis 1: 63.3% of variance based on r^2^, Axis 2: 19.3% of variance). Countries belonging to the same language family (Romanic- or Slavic) group together, showing that they have a similar culinary tradition of edible mushrooms. However, Slavic-speaking countries are clearly separated into Eastern European countries and SE European countries. Neighbouring countries such as Belgium, Switzerland and France, influence each other, especially if there is a cultural exchange between them, e.g. due to a common language. Austria, the only Germanic-speaking country with mushroom guidelines or legislation, has an intermediate position. We speculate that this may reflect the situation of a country with a predominantly mycophobic population, but with a vivid cultural exchange with both its Romanic and Slavic neighbours.

A very interesting aspect of this work was to discover typical, distinctive culinary preferences of Slavic or Romanic speaking people, of people from special geographical regions or from different zones: such indicator species or indicator genera reflect both, the different taste of the mushroom consumer, and the different know-how of mushroom cooking and preservation. Moreover, different collecting habits (e.g. collecting hypogeous fungi with the help of dogs or pigs) can also be differentiated.

Romanic-speaking people living in the west of Europe collect and consume significantly more species of the genera *Agrocybe* and *Amanita* than Slavic-speaking people. Moreover, Western Europeans can be distinguished from other mushroom consumers by their love for mushrooms belonging to the genera *Coprinus*, *Craterellus*, *Flammulina*, *Morchella*, *Macrolepiota*, *Stropharia, and Tricholoma* (Tables 4–5). In Eastern Europe the population likes to consume *Suillus spp.*, while the population from the Nordic Co-operation, besides *Suillus variegatus* (Velvet Bolete), appreciates several species of *Russula* (*R. claroflava* - Yellow Swamp Brittlegill, *R. decolorans* - Copper Brittlegill, *R. vinosa* - Darkening Brittlegill), *Leccinum vulpinum* (Foxy Bolete) and *Hygrophorus camarophyllus* (Arched woodwax). Several exotic species of boletes can be traded in countries in the SE of Europe, a distinctive feature of these markets (Table 5).

**Table 4:** List of indicator genera of wild edible mushroom for language families (Romance), for neighbouring countries (West, East) or for geography (Central Europe, SW-Europe).

Indicator genus	Category	Group	p^a^	
*Amanita*	Geography	SW Europe	0.0170	*
*Flammulina*		Central Europe	0.0130	*
*Stropharia*		Central Europe	0.0128	*
*Agrocybe*	Language family	Romance	0.0138	*
*Amanita*		Romance	0.0048	**
*Agrocybe*	Neighbours	West	0.0002	***
*Coprinus*		West	0.0072	**
*Craterellus*		West	0.0012	**
*Flammulina*		West	0.0136	*
*Grifola*		West	0.0104	*
*Lepista*		West	0.0064	**
*Macrolepiota*		West	0.0002	***
*Morchella*		West	0.0014	**
*Stropharia*		West	0.0126	*
*Tricholoma*		West	0.0010	**
*Suillus*		East	0.0022	**

a Only significant p-values <0.02 are shown.

* p≤0.05;

** p≤0.005;

*** p≤0.0005.

**Table 5:** List indicator species of wild edible mushrooms for neighbouring countries (East, West, Former Yugoslavia) and for geography (SW-Europe, SE Europe, Nordic Co-operation).

Indicator species	Category	Group	p^a^	*
*Hygrophorus camarophyllus*	Geography	Nordic Co-operation	0.0014	**
*Leccinum vulpinum*		Nordic Co-operation	0.0050	**
*Russula claroflava*		Nordic Co-operation	0.0022	**
*Russula decolorans*		Nordic Co-operation	0.0022	**
*Russula vinosa [ = R. obscura]*		Nordic Co-operation	0.0022	**
*Suillus variegatus*		Nordic Co-operation	0.0034	**
*Boletus fechtneri*		SE Europe	0.0092	**
*Boletus impolitus*		SE Europe	0.0092	**
*Boletus pulverulentus*		SE Europe	0.0092	**
*Boletus rhodoxanthus*		SE Europe	0.0092	**
*Boletus torosus*		SE Europe	0.0092	**
*Tricholoma terreum*		SW Europe	0.0014	**
*Leccinum aurantiacum*	Neighbours	East	0.0062	**
*Suillus bovinus*		East	0.0060	**
*Suillus luteus*		East	0.0014	**
*Suillus variegatus*		East	0.0018	**
*Xerocomus subtomentosus*		East	0.0030	**
*Agaricus campestris*		West	0.0002	***
*Agrocybe cylindracea*		West	0.0010	**
*Coprinus comatus*		West	0.0070	**
*Craterellus lutescens*		West	0.0004	***
*Craterellus tubaeformis*		West	0.0092	**
*Flammulina velutipes*		West	0.0096	**
*Hericium erinaceus*		West	0.0096	**
*Morchella elata*		West	0.0026	**
*Tuber macrosporum*		Former Yugoslavia	0.0026	**

a Only highly significant p-values <0.001 are shown.

* p≤0.05;

** p≤0.005;

*** p≤0.0005.

Indicator species also permitted us to test our hypothesis, e.g. that the detected mushroom preference is not only related to the fungal distribution (which often follows the distribution of ectomycorrhizal host plants and is therefore climate-related). Most of the detected indicator species, e.g. *Suillus variegatus* and *Russula decolorans* have a broad geographical range, and therefore clearly indicate culture-specific preferences (Slavic, Nordic Co-operation) for these mushrooms.

When considering collecting habits or know-how of mushroom preparation, Romance-speaking people in the west of Europe, for instance, collect morels during spring, an unusual season for mushroom picking; moreover, people from Northern Europe know how to prepare *Russula* spp. with an acrid taste, and in turn these are considered to be uneatable in Romance-speaking countries.

### Mushroom guidelines or legislation are in flux

Mushroom guidelines or legislation have been and are being changed and adapted all the time: they must incorporate new scientific findings, and they have to meet changes to risk requirements or a change in the consumption behaviour of the population. Advice or statements on mushroom edibility are often based on traditions, on empirical experience based on mixed mushroom dishes, but not on toxicological risk assessment. Based on new scientific findings, several fungal species have recently been removed from some European lists of edible mushrooms: the most striking case concerns cases of massive rhabdomyolysis, removed since 1993 in France and 2001 in Poland. This new mushroom intoxication syndrome occurred after the ingestion of large amounts of an edible and, until then, valuable species called *Tricholoma equestre* [17]. Thereupon, *T. equestre*, *T. flavovirens* and the closely related *T. auratum* were removed from most European edible mushrooms lists. *Amanita ovoidea*, *Clitocybe nebularis*, *Coprinopsis atramentaria*, *Gyromitra* spp., *Laccaria amethystina*, *Paxillus involutus* and *Ramaria formosa* also contain toxicants, and are therefore suspected of causing acute or long-term adverse effects after ingestions [7], [8], [18], [19], [20]. The Honey Fungus (*Armillaria mellea* s.l.) should only be consumed when thoroughly cooked; moreover, one species of this taxonomically difficult complex (*A. ostoyae*) is toxic [8], [21]. *Lactarius torminosus* has also been considered to be toxic [22]. Despite these reports of toxicity, fruit bodies of *L. torminosus* mushrooms are prepared in Finland, Russia, and other Northern and Eastern European countries by parboiling, soaking in brine for several days, or pickling, after which it is highly valued for its peppery taste. False Morels (*Gyromitra* spp.) were among the most popular wild mushrooms sold in markets in Finland [23]. They could be sold in the market place or in supermarkets, but there had to be a label warning that these mushrooms were very poisonous if not specially treated: they needed to be either boiled twice for 10 minutes, each, in 10 litres of water/1 kg of *Gyromitra* spp., or to be dried properly and then boiled. In 2012, the Finnish mushroom legislation was supplemented with the “Guidance lists on mushrooms” of the Nordic Co-operation [7], [8].

## Conclusions

### Mushroom legislation or guidelines as a consequence of tradition and practice

The commerce of fresh and preserved wild mushrooms is very important in countries with a mycophilic population. This is reflected by a comparatively large number of mushroom species on their lists for the commercialisation of wild mushrooms, and by the large number of poisoning cases in mycophilic cultures [1], [3], [17], [24], [25]. To minimise this unpleasant side effect of mushroom consumption, mycophile countries have usually released guidance lists or legislation concerning the commerce of wild mushrooms. Mushroom guidelines or legislation are thus based on practical necessities and the current use of edible mushrooms in a country. They contain only species, which are collected, traded and consumed by the local population. These mushroom species are also usually broadly known among the population, and there is a general know-how concerning how, when and where to collect them, and concerning their safe preparation. The richest market in terms of species diversity ever documented was undoubtedly the one of Trento (Italy), where more than 250 mushroom species were observed on sale [26] before national Italian legislation limited the menu of species in 1955 [4]. The richest markets can nowadays be found in France, Switzerland and Spain.

Mushroom commerce is not a topic of general interest in countries with a mycophobic population. Only a few species of edible mushrooms are usually traded in such countries, most of them being cultivated or imported. However, food harvested from the wild including mushrooms, is of a growing interest in most countries. Therefore, guidelines based on risk assessment are also of importance for countries with a mycophobic population, especially because there is less appropriate knowledge on a safe consumption of wild mushrooms. Such guidelines guarantee food safety, and are therefore especially important for mushroom trade.

### Consequences for the mushroom trade and trade of mushroom products in Europe

Mushrooms can be a big business: 100 metric tons of fresh mushrooms were sold per year alone in the city of Milano from 1919 onward [27]. In the nineties, the estimated world production of wild chanterelles (*Cantharellus* spp.) ranged from 150 000 to 200 000 metric tons per year, with a value of about $ 1.7 billion in the market place [19]; the worldwide supply of black truffles was estimated at more than 200 metric tons per year, with an estimated world market value of probably not more than1 billion SEK; furthermore, Italy alone imported 54 557 metric tons of mushrooms, and exported 5 487 metric tons in 2005, with ever increasing imports from Asia, the Balkans and from Slavic countries [4]. China was the largest mushroom producer with 22% of the worldwide production in 2004. The largest mushroom producers in Europe at that time were The Netherlands, France, Poland and Spain, accounting for 20% of the worldwide mushroom production [28].

Mushroom pickers sell their bounty to local restaurants and foreign markets. New markets are opening up all the time, and in consequence of globalisation the least popular species consumed in countries with a mycophobic population are likely to rise. Moreover, new global trends such as “sustainable eating” or “healthy eating” change consumption behaviour, bringing more mushrooms and mushroom products onto the consumer's plate. However, the processes of internationalisation and cultural homogenisation can also result in a reduced diversity and in changing positions of mushroom species in gastronomy: wild mushrooms are very important in Italian culinary tradition. Wild mushroom markets have therefore flourished in Italy for centuries, but preferences and consumption behaviour were regionally very different before the 20^th^ century. From then on, Italy has emerged as a focal point of a global market for a small number of mushroom species, especially porcini. This has caused nationalisation in culinary fashion, coming at the expense of differing, localised mushroom traditions [4].

### Do we need a European guidance/legislative list for wild mushrooms?

The five top-selling edible fungi (*Boletus edulis* species group, *Cantharellus cibarius*, *Lactarius deliciosus*, *Morchella esculenta*, and *Agaricus campestris*) are listed in most European mushroom lists. The most important wild mushroom species currently traded in Italy apart from these top-sellers are St. George's Mushroom (*Calocybe gambosa*) and Honey fungi (*Armillaria mellea* s.l.), mushrooms, which are not on the list of several European countries. However, articles 23–30 of the European Union treaty guarantee the free movement of goods within the EU, which is not consistent with being able to sell a mushroom species in one country, but not in another [4]. On the other hand, the European Union treaty requests food safety, so it fits well with the fact that a mushroom could be sold in a country where the population has specific knowledge about the preparation of this species, but not in another country where such general knowledge or traditions are lacking. E.g. False Morel cannot be sold in Denmark, but can be sold in Finland.

It would make sense if the European Commission evaluated individual national legislation on edible mushrooms. In doing so it could propose education, identification and safety evaluation, and perhaps draft guidelines and legislation for edible and, maybe more importantly, for potentially toxic species. A European guidance list including all edible mushroom species currently commercialised in European countries could be a meaningful, foundation for national lists, which include a selection of species: most of our 268 fungal species on the list only have local significance as edible mushrooms: half of the mushrooms are sold in one or two European countries only. National lists rely on a traditional mycological knowledge present in the population of one cultural group, but not present in another. Local market dealers must have basic skills in mushroom identification, as well as a knowledge of any special treatment required by certain species before consumption [29]. Nevertheless, mushroom lists containing too many species are very difficult to manage, because they require a large number of well-trained mycologists controlling the fungi on the markets. It was therefore not surprising that Switzerland reduced the number of edible mushroom species from 142 to 114 in 2012. Based on the situation in European countries, about 60 edible mushroom species seem to be appropriate for a national mushroom legislation.

## Supporting Information

Table S1 References for information concerning legislation or guidelines on the commercialisation of wild mushrooms in 42 European countries.
(DOCX)

Table S2 Edible fungal species listed in European countries concerning the commercialisation of wild mushrooms. The list also provides information whether the mushroom can be cultivated or not, on the taxonomic affiliation and on the percentage of European lists where since 2012. Potentially toxic fungal species are marked (+). The list includes also the old Switzerland list valid until 2012. This latter list was not used for analyses, but is for information only. The total number of edible mushrooms species on the respective list is provided in the second line, n = 268, but n = 282 until Switzerland changed the list in 2012. Abbreviations: A: Ascomycota, B: Basidiomycota, B_A: B_Agaricales, B_Au: B_Auriculariales, B_B: B_Boletales, B_G: B_Gasteromycetes, B_C: B_Cantharellales, B_P: B_Poroid, B_R: B_Russulales, B_T: B_Tremellales.
(XLSX)

Table S3 Fungal genera reported in 22 European lists concerning the commercialisation of mushrooms. The number of species of the respective genus allowed for a specific country is provided. The list also provides information whether the mushroom can be cultivated or not, and on the taxonomic affiliations. Abbreviations: A: Ascomycota, B: Basidiomycota, B_A: B_Agaricales, B_Au: B_Auriculariales, B_B: B_Boletales, B_G: B_Gasteromycetes, B_C: B_Cantharellales, B_P: B_Poroid, B_R: B_Russulales, B_T: B_Tremellales.
(XLSX)
