# Supplementary material for: Mycophilic or Mycophobic? Legislation and Guidelines on Wild Mushroom Commerce Reveal Different Consumption Behaviour in European Countries
Source: PLoS One. 2013 May 21;8(5):e63926. doi: 10.1371/journal.pone.0063926 (PMC3660358; doi:10.1371/journal.pone.0063926)
Supplement: Table S1 — References for information concerning legislation or guidelines on the commercialisation of wild mushrooms in 42 European countries. (DOCX) [file pone.0063926.s001.docx]

| **Legislation / guidelines** | **Country** | **Reference** |
| --- | --- | --- |
| **With Legislation** | Austria | Bundesgesetzblatt für die Republik Österreich, 386. Verordnung: Speisepilzverordnung, 1997 http://faolex.fao.org/cgi-bin/faolex.exe?rec_id=007878&database=FAOLEX&search_type=link&table=result&lang=eng&format_name=@ERALL |
|  | Belarus | Санитарные правила и нормы 2.3.4.13-6-2004, ГИГИЕНИЧЕСКИЕ ТРЕБОВАНИЯ К ЗАГОТОВКЕ, ПЕРЕРАБОТКЕ И ПРОДАЖЕ ГРИБОВ (Appendix 1. Table 1. List of mushrooms, allowed to stocking and included in the standards of mushroom production. In: Sanitary regulations and norms 2.3.4.13-6-2004: Hygiene demands for stocking, treatment and selling of mushrooms [Ministry of Health Care of the Republic of Belarus; Republican sanitary-hygienical and sanitary-antiepidemic regulations and norms]. Minsk, 2004. P. 28-37. (44 pp.) [<http://www.minzdrav.gov.by/med/docs/tnpa/Gigiena_233-9_2004_izm2009.doc>](http://www.minzdrav.gov.by/med/docs/tnpa/Gigiena_233-9_2004_izm2009.doc) ) |
|  | Bosnia and Herzegovina (including Republika Srpska) | Pravilnik o uzgoju, iskorištavanju, sakupljanju i prometu sekundarnih šumskih proizvoda. 2005. Službene novine FBiH 66/05. [Rulebook on Growing, Use, Collection and Marketing of Secondary Forest Products. 2005. Official Gazette FBiH no. 66/05] <http://www.fmpvs.gov.ba/texts/206_48_b.pdf>. Pravilnik o uvjetima korištenja ostalih šumskih proizvoda i načinu sakupljanja njihovih nadoknada. 2010. Službeni glasnik Republike Srpske 8/2010. [Ordinance on the use of other non-timber forest products and the procedures for the obtaining of compensation for their exploitation. 2010. Official Gazette of the Republika Srpska no. 8/2010] http://docs.slglasnik.org/slgl/show_docs.jsf |
|  | Croatia | Pravilnik o zaštiti gljiva. 2002. Narodne novine 34/2002. [Ordinance on Protection of Fungi. 2002. Official Gazette no. 34/2002] (Tkalčec et al. 2003) |
|  | Finland | List of Mushrooms Suitable for Marketing“ (3/2007) based on Decree of Marketing of Mushrooms (498/2006) given by the Ministry on Trade and Industry. This list was valid until July, 1, 2012 and is now complemented by the Guidance list of Nordic cooperation [[7](#_ENREF_7),[8](#_ENREF_8)]. |
|  | France | Anonyme 2012. Décret n° 2012-129 du 30 janvier 2012 relatif à la mise sur le marché des truffes et des denrées alimentaires en contenant. Journal Officiel de la République Française 31/01/2012, texte n° 28. NOR : EFIC1134213D. Anonyme 2005. Décret n° 2005-1184 du 19 septembre 2005 portant interdiction de plusieurs espèces, sous-espèces ou variétés de champignons. Journal Officiel de la République Française 21/9/2005: 15187, texte n° 9 [[5](#_ENREF_5)]. |
|  | Italy | La legislazione italiana in materia di raccolta e commercializzazione dei funghi epigei freschi e conservati. Normativa quadro in materia di raccolta, coltivazione e commercio die tartufi freschi o conservati destinati al consumo (1985). Regolamento concernente la disciplina della raccolta e della commercializzazione dei funghi epigei freschi e conservati (1995). Lista nazionale ALLEGATO I – Lista positiva delle specie di funghi epigei spontanei commercializzabili allo stato fresco. Raccolta di leggi per i corsi di formazione delle Guardie Ecologiche Volontarie (2001), http://www.salute.gov.it/sicurezzaAlimentare/paginaInternaMenuSicurezzaAlimentare.jsp?id=1174&lingua=italiano&menu=funghi |
|  | Macedonia | Spisok na zagrozeni vidovi gabi. 2004. Služben Vesnik na Republika Makedonija 91/2004. [List of Threatened Species of Fungi. 2004. Official Gazette of the Republic of Macedonia no. 91/2004] http://www.slvesnik.com.mk/Issues/9A41AC933DC49B4DB26C644B4C89C17A.pdf  Listi za utvrduvanje na strogo zaštiteni i zaštiteni divi vidovi vo Republika Makedonija. 2011. Služben Vesnik na Republika Makedonija 139/2011. [Lists for Designation of Strictly Protected and Protected Wild Species in the Republic of Macedonia. 2011. Official Gazette of the Republic of Macedonia no. 139/2011] http://www.slvesnik.com.mk/Issues/BEA371DCFB672E4894ACA18E9D04ADD1.pdf |
|  | Montenegro | Pravilnik o bližem načinu i uslovima sakupljanja, korišćenja i prometa nezaštićenih divljih vrsta životinja, biljaka i gljiva koje se koriste u komercijalne svrhe. 2010. Službeni list Crne Gore 62/2010. [Ordinance on the detailed procedures and conditions of collecting, using and trading of unprotected wild species of animals, plants and mushrooms used for commercial purposes. 2010. Official Gazette of the Republic of Montenegro no. 62/2010] http://www.sluzbenilist.me/PravniAktDetalji.aspx?tag={216B26C8-06F7-4F5C-957D-13076A6B0AD7} |
|  | Poland | Rozporzndzenie Ministra zdrowia, z dnia 13 listopada 2008 r. http://www.pfpz.pl/index/?id=cebd648f9146a6345d604ab093b02c73 |
|  | Romania | Ordin nr. 246 din 14 aprilie 2006 pentru stabilirea Listei ciupercilor comestibile din flora spontana a caror recoltare sau achizitie si comercializare sunt permise. 2006. Monitorul Oficial nr. 367/2006. [Ordinance no. 246 of 14 April 2006 on establishing the list of wild edible fungi allowed for picking or purchasing and selling. 2006. Official Gazette 367/2006] http://legestart.ro/Ordinul-246-2006-stabilirea-Listei-ciupercilor-comestibile-flora-spontana-caror-recoltare-achizitie-comercializare-sunt-permise-%28MTg5OTcz%29.htm |
|  | Russia | Victor Mukhin, translation of the “Official List of EDIBLE MUSHROOMS authorized for marketing or production of mushroom preserves, and foodstuffs containing mushroom in Russia”<http://www.kadis.ru/texts/index.phtml?id=50029> |
|  | Serbia | Uredba o izmenama i dopunama Uredbe o stavljanju pod kontrolu korišćenja i prometa divlje flore i faune. 2010. Službeni glasnik Republike Srbije 09/2010. [Ordinance on the amendments to the Ordinance on the imposing of control on the use and trade of wild flora and fauna. Official Gazette of the Republic of Serbia no. 09/2010] (Ivančević et al. 2012) |
|  | Slovakia | Potravinový kódex SR [Food Codex of the Slovak Republic] http://www.svps.sk/legislativa/legislativa_kodex.asp III. časť [3rd part] |
|  | Sweden | <http://www.slv.se/sv/grupp1/Risker-med-mat/Svamp-och-svampgifter/Lampliga-matsvampar/> |
|  | Spain | Real Decreto 30/2009, de 16 de enero and Moreno & Arregu 2002 |
|  | Switzerland | Verordnung über Speisepilze (Pilzverordnung, VSp), 2002 <http://www.admin.ch/ch/f/as/2002/781.pdf> revised in 2012: <http://www.admin.ch/ch/f/rs/817_022_106/app1.html#ahref1> |
| **With Guidelines** | Belgium | l'Arrêté royal relatif à la fabrication et au commerce de denrées alimentaires composées ou contenant des plantes ou préparations de plantes (1997, pp. 24-26) <http://www.bbkbio.be/pdf/KB290897_geconsolideerde-versie-op-28022005_FR.pdf> |
|  | Nordic cooperation | Mushrooms traded as food Nordic questionnaire, including guidance list on edible mushrooms suitable and not suitable for marketing Nordic Council of Ministers 2012 [[7](#_ENREF_7),[8](#_ENREF_8)]. Nordic cooperation= Denmark, Finland, Iceland, Norway, Sweden, Faroe Islands, Greenland, Aland |
|  | Portugal | Baptista-Ferreira J. L., personal communication based on a conservative estimation of current use in the country [[10](#_ENREF_10)]. |
|  | Ukraine | Vera Hajova based on Zerova et al. [[9](#_ENREF_9)]. |
| **No lists** | Bulgaria | Cvetomir M. Denchev, personal communication. |
|  | Estonia | Irja Saar, personal communication. |
|  | Germany | Reinhard Agerer, Frank Dämmrich, personal communication. |
|  | Great Britain | Sally Evans, Andy Taylor, personal communication. |
|  | Greece | Stefanos Diamandis, personal communication. |
|  | Hungary | László Jáger, personal communication. |
|  | Irleland | Sally Evans, personal communication. |
|  | Lithuania | Jonas Kasparavičius, personal communication. |
|  | Latvia | Inita Daniele, personal communication. |
|  | Netherlands | Eef Arnolds, personal communication. |
|  | Slovenia | Uredba o varstvu samoniklih gliv. 1998. Uradni list Republike Slovenije 57/1998. [Decree on the Protection of Wild Fungi. 1998. Official Gazette of the Republic of Slovenia 57/1998] <http://www.uradni-list.si/1/content?id=7610>, Uredba o zavarovanih prosto živečih vrstah gliv. 2011. Uradni list Republike Slovenije 58/2011. [Decree on Protected Species of Wild Fungi. 2011. Official Gazette of the Republic of Slovenia 58/2011] http://www.uradni-list.si/1/objava.jsp?urlid=201158&stevilka=2723 |
